# Four New Species and New Records of Orbilia from China Based on Molecular and Morphological Data

**DOI:** 10.3390/jof8111188

**Published:** 2022-11-11

**Authors:** Xiao-Yun Ou, Yuan-Yuan Shao, Hai-Fu Zheng, Bin Liu

**Affiliations:** 1 Institute of Applied Microbiology, College of Agriculture, Guangxi University, Nanning 530005, China; 2 Key Laboratory of Beibu Gulf Environment Change and Resources Utilization of Ministry of Education, Nanning Normal University, Nanning 530001, China; 3 Guangxi Forest Inventory & Planning Institute, Nanning 530011, China

**Keywords:** *Orbilia*, new species, morphology, phylogenetic, taxonomy

## Abstract

This study reports four new species and three new record species of Orbiliaceous fungi from China. *Orbilia baisensis*, *O. hanzhongensis*, *O. nanningensis* and *O. pinea* are described as new species and *O. crenatomarginata*, *O. vinosa* and *O. vitalbae* are described as new record species. All the studied species were identified by morphological characteristics and phylogenetic analysis of internal transcribed spacer (ITS) and large subunit (LSU) sequences. Four new species are described based on their sexual and asexual states, and their differences with the close relatives were compared and discussed.

## 1. Introduction

The family *Orbiliaceae* is characterized by producing tiny, waxy, translucent, light-colored, sessile to sub-stipitate apothecia with small ascospores, which are asymmetrically globose to sub-fusoid [1]. Members of family *Orbiliaceae* are widely distributed in the environment and sporadically in arid habitats as saprophytic, parasitic or superficial on tree bark, deadwood, withered leaf and animals’ excrement [2,3]. The most prominent feature of the family *Orbiliaceae* is the presence of a plasmatic spore body which is a strongly refractive vacuolar in the ascospore and is only visible in the living state [4]. The genus of *Orbilia* was established to accommodate *Orbilia leucostigma* [5], and the family *Orbiliaceae* was recognized by Nannfeldt [6] and assigned to the order Helotiales, which was revised to the class Leotiomycetes [4,7]. Attributed to the morphological features and molecular phylogenetic evidence, *Orbiliaceae* was transferred to the order *Orbiliales* and the class *Orbiliomycetes*, comprising two teleomorphic genera, *Orbilia* Fr. and *Hyalorbilia* Baral [8]. The third teleomorphic genus *Pseudorbilia* includes only one species carrying the characteristics intermediate between *Orbilia* and *Hyalorbilia* [9,10]. In the past decade, there is a continuous documentation of new species and taxonomic reforms, depicting the evolutionary changes in the diversity of the genus *Orbilia* [1,4,11,12,13,14,15,16,17,18,19,20]. Seven sexual-type genera and three asexual-type genera are accepted in the family *Orbiliaceae*; the teleomorph-typified include *Amphosoma*, *Bryorbilia*, *Liladisca*, *Lilapila*, *Pseudorbilia*, *Hyalorbilia* and *Orbilia*, while the anamorph-typified include *Lecophagus*, *Mycoceros*, *Retiarius*, and 415 species of *Orbilia* have been assigned among these genera [3].

The internal transcribed spacer (ITS) [21] and the large subunit gene of the rDNA (LSU) [22] region are extensively employed in phylogeny studies of fungi and these two markers have been proven to be effective to study the phylogenetic relationship in Orbiliaceous fungi. More recently, in order to overcome the ambiguities associated with *Orbilia leucostigma* and *Orbilia xanthostigma*, Baral revealed the high variation of ITS and LSU and presented distinct genotypes [23]. However, some studies have also made use of several other genes, which include the translation elongation factor 1-alpha (TEF) [24], beta-tubulin (TUB) [25,26], RNA polymerase second largest subunit (RPB2) [27,28] and chitin synthase 1 (CHS-1) [29], etc.

The concept of sexual and asexual states in Orbiliomycetes was first established by Brefeld [30] but it was well explained by Pfister [31]. The asexual states of *Orbiliaceae* include *Arthrobotrys*, *Dactylella*, *Dactylellina*, *Dicranidion*, *Drechslerella*, *Helicoon*, *Tridentaria*, *Trinacrium*, etc., while the *Arthrobotrys*, *Dactylellina* and *Drechslerella* belong to nematode-trapping fungi. Harkness established *Dicranidion* based on *Dicranidion fragile* Harkness [32], while the genus *Dicranidion* was placed into the section 2 of Hyphomycetes by Hughes [33]. The conidia of *Dicranidion* consist of two or three lobes, and in some species, conidia have multiple lobes, the lobes are equal or unequal, parallel or unparallel, septate or non- septate [34]. Brefeld firstly reported *Dicranidion* sp. that isolated from *Orbilia*, Berthet described the conidia of *Orbilia xanthostigma* with illustrations [35].

China is rich in endemic species resources and biological diversity owing to its varying environmental and geographic regions. During surveys of the orbiliaceous fungi from Guangxi Province of Southwestern China and Shaanxi province of Northwestern China, seven species of *Orbilia* were found and identified based on morphological evidence together with LSU and ITS sequence data. Among them, *Orbilia baisensis*, *Orbilia hanzhongensis*, *Orbilia nanningensis* and *Orbilia pinea* are described as new species, and *Orbilia crenatomarginata*, *Orbilia vinosa* and *Orbilia vitalbae* are described as new Chinese record. 

## 2. Materials and Methods

### 2.1. Morphological Studies

Fresh specimens were collected from decayed and fallen tree branches and wood logs from Guangxi and Shaanxi provinces, China. In the description, the symbols were adopted as follows: * = living state, † = dead state. The specimens were dried and deposited in the GXU (Herbarium of Institute of Applied Microbiology, Guangxi University, China).

To obtain a pure culture, a fresh apothecium was fixed to the lid of a petri dish with the hymenia facing downward, allowing the ascospores to shoot on the surface of the water agar (18 g agar, 1 L distilled water). After germination, the ascospores deposits were transferred onto PDA (Potato Dextrose Agar) plates [36], MEA (Malt Extract Agar) plates, CMA (Corn Meal Agar) plates and LY (Lactose-Yeast Extract Agar) plates and incubated for 5–10 days at 25 °C. Cultures was deposited in the Institute of Applied Microbiology, Guangxi University, China. Observations and photographs were taken with a Nikon Eclipse 80i microscope (Nikon Corporation, Tokyo, Japan) equipped with Nikon Digital Sight DS-L1 microphotographic system. All the morphological measurements were recorded from 20 elements in water mounts employing Spot32 software v4.0.8 (Diagnostic Instruments, Sterling Heights, MI, USA).

### 2.2. DNA Extraction, PCR Amplification and Sequencing

Mycelia from the fresh cultures were inoculated in the potato dextrose broth (PDB) and were cultured under dark conditions in a thermostable shaker at 25 °C. After 2 weeks of shaking, mycelia were collected and washed with sterile distilled water and were used to extract DNA by CTAB method [37]. For those species without pure cultures, the sequencing DNA was directly amplified from the hydrated apothecia as described by Vitória et al. [38]. Briefly, the apothecia were placed in a PCR tube using a needle and stored at −80 °C for 12–24 h. One apothecium was transferred to a PCR tube containing 3 μL Cell Lysis Buffer, and vortexed for 2 min at maximum speed followed by incubation at 80 °C for 15 min. The samples were preserved at −20 °C for later use or directly used for PCR amplification. 

Sequence data were generated from the internal transcriber spacer region of nuclear ribosomal DNA (ITS) and the large subunit of the rDNA genes (LSU) using primer pairs ITS1/ITS4 [21] and LROR/LR5 [22]. PCR amplification was performed in a reaction mixture of 50 μL containing 25 μL 2X Taq-Plus PCR Master Mix, 1 μL of each primer, 22 μL of doubly distilled H_2_O, 1 μL of DNA template and the total. PCR reaction conditions were as follows: initial denaturation at 94 °C for 5 min; followed by 35 cycles of denaturation at 94 °C for 1 min, annealing at 56 °C for 1 min and extension at 72 °C for 1 min; and a final extension at 72 °C for 5 min. Amplified PCR products were separated on 1% agarose gel and examined under the UV light. PCR products were sequenced from the Beijing Genomics Institute (BGI).

### 2.3. Phylogenetic Analysis

Thirteen new sequences were generated in this study. To establish the preliminary identification of the studied species, the acquired sequences were first carefully examined for intactness and then blasted in the NCBI nucleotide sequence blast and were compared with the already published data. The obtained sequences of ITS and LSU were then used for phylogenetic studies. Related sequences of similar species were downloaded from NCBI GenBank and the sequences data sets were aligned using Clustal X 1.83 [39] and converted to FASTA files and constructed maximum likelihood tree by MEGA version 6.06 using the Kimura 2-parameter model [40]. The sequences were converted to NEXUS files by Phylosuite [41] and the partition homogeneity test was performed with 1000 replicates in PAUP*4 [42]. Nucleotide substitution models were selected by MrModeltest 2.31 [43]. The corresponding phylogenetic trees were constructed using the maximum likelihood and Bayesian inference analyses. Maximum likelihood analyses were performed with MEGA version 6.06, and Bayesian inference analyses were carried out using MrBayes v3.2.2 [44]. The tree was viewed in Fig Tree v1.4.4 [45]. The maximum likelihood bootstrap proportions (MLBP) were above 50% and Bayesian inference posterior probabilities (BIPP) greater than 0.95 at nodes. GenBank accession numbers are given in Table 1.

## 3. Results

### 3.1. Taxonomic Description

#### 3.1.1. New Species

**Figure 1:**
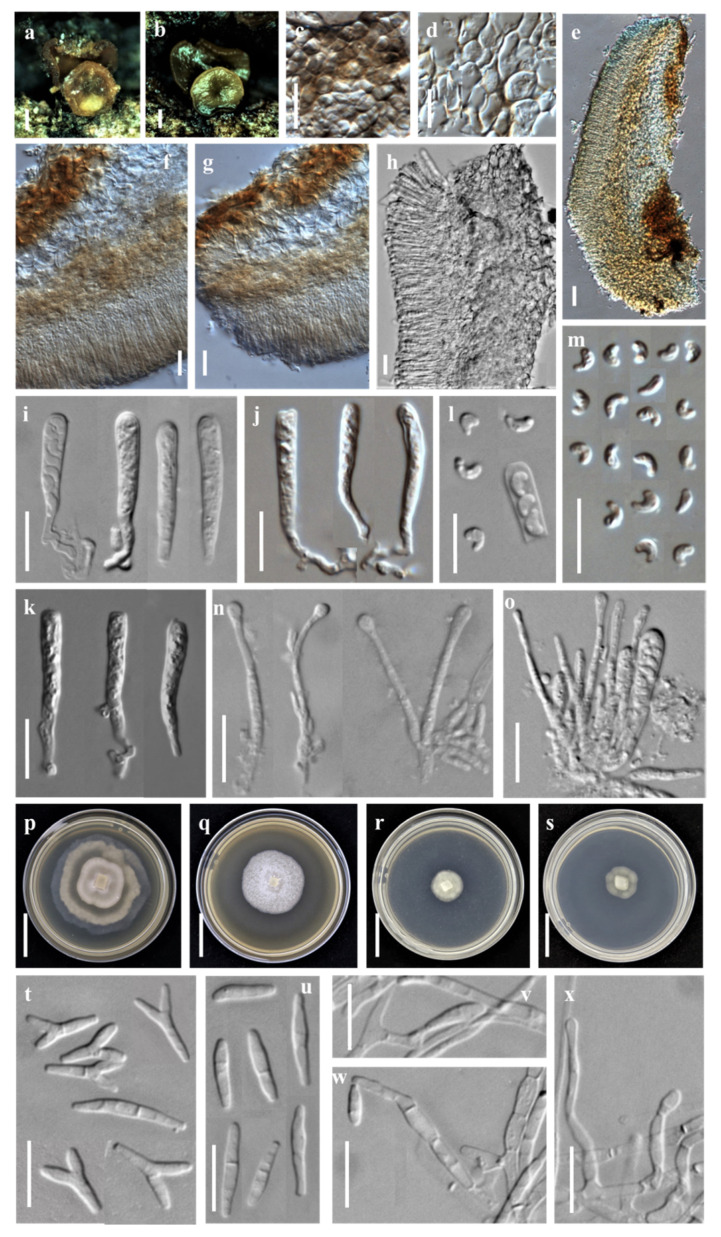
*Orbilia baisensis* and *dicranidion*-like asexual morph (strain DL17 was isolated from GXU2279). (**a**,**b**) apothecia; (**c**,**d**) basal excipular cells; (**e**–**h**) vertical section of apothecium; (**i**–**k**) ascus; (**l**,**m**) ascospores; (**n**) paraphyses; (**o**) asci and paraphyses; (**p**–**s**) colony after 10 d at 25 °C, (**p**) on PDA, (**q**) on MEA, (**r**) on CMA, (**s**) on LY; (**t**,**u**) conidia; (**v**–**x**) conidiophores with conidia. Scale bars: (**a**,**b**) = 0.5 mm; (**c**,**d**,**f**,**g**,**i**–**o**,**t**–**x**) = 10 μm; (**e**,**h**) = 20 μm; (**p**–**s**) = 10 mm.

***Orbilia baisensis*** X.Y. Ou and Bin Liu, sp. nov. (Figure 1).

MycoBank: MB 846093.

Etymology: from the geographical origin, Baise (Guangxi).

Holotype: CHINA, Guangxi province, Baise city, Dawangling drift scenic spot, from deadwood of *Castanea mollissima* on the ground, 11 July 2016, X.Y. Ou, GXU2279. Strain DL17 was isolated from GXU2279.

Sexual state: Apothecia superficial on the deadwood of *Castanea mollissima*, 0.2–1.4 mm in diameter, gregarious in groups or scattered, waxy, translucent, smooth, disc slight concave to flat, margin not protruding, sessile, yellow when fresh or rehydrated, turned yellow to orange when dry. Asci †12.2–39.3 × 2.3–4.6 μm, cylindric-clavate, 8-spored, pars sporifera †18.5–23.6 μm long, truncate to hemispherical at the apex, gradually narrowing towards to the base, flexuous stalk, forked to L-, T- or Y-shaped. Ascospores †2.9–4.6 × 1.6–2.1 μm, hyaline, non-septate, smooth, pronounced reniform, strongly curved ~90–150°, one end round to obtuse, the other end small with a short pointed base, pairs of ascospores arranged in the ascus; containing one refractive globose SB (spore body) at the end close to the wall in alive mature ascospores, 0.6–1.1 μm diameter. Paraphyses apically inflated to mostly capitate (-clavate), †21.9–29.0 × 0.8–1.9 μm, basally branched and expanding to 1.8–3.1 µm in diameter at the apex. Hymenium 70.5–192.3 μm thick; medullary excipulum 25.8–40.6 μm thick, of ± loose textura intricata with ± inflated cells, sharply delimited. Ectal excipulum composed of textura globulosa-angularis, thin-walled to slightly gelatinized, 38.8–146.6 μm thick, cells †3.2–7.9 × 2.0–5.5 μm diam., ovate to spherical.

Asexual state: *Dicranidion*-like.

Colonies beige-white on PDA, 34.8 mm in diameter at 25 °C after 10 d, strongly keratinized, aerial hyphae absent; beige-white on MEA, 24.8 mm diam., aerial hyphae sparse; white on CMA and LY, 11.8–12.0 mm diam., aerial mycelium absent; Hyphae hyaline, septate, branched, smooth and 1.4–3.2 μm wide. Conidiophores hyaline, erected or slightly bent, septate, unbranched, *5.7–33.1 μm long, *2.0–2.8 μm wide at the base and gradually tapering to *0.9–1.5 μm wide at the tip where bearing 1 apical spore. Conidia thallic, hyaline, Y-shaped, consisted of a spindle and two equal or unequal lobes; the spindle *6.0–11.5 μm long, 1–2 septate; the lobe *2.0–6.7 × 1.5–2.3 μm, 1 septate; in addition, columnar conidia *6.3–20.0 × 1.8–2.6 μm, 1–3 septate.

Additional specimen examined: China, Shaanxi province, Hanzhong city, Foping County, Wangjiawan, from deadwood, 23 July 2017, X.Y. Ou, GXU2373.

Notes: *Orbilia baisensis* is clustered with *O. renispora* Y.Y. Shao, Quijada, Baral, Haelew. and Bin Liu, *O. leucostigma* (Fr.) Fr., *O. xanthostigma* (Fr.) Fr. and *O.* cf. *xanthostigma* (as *O. delicatula*) by having reniform to nephroid or C-shaped ascospores and their asexual states are belonging to *Dicranidion*. However, *O. baisensis* features on 8-spored asci, flexuous base and forked to L-, T- or Y-shaped, pronounced reniform ascospores, round and larger at one end, the other end with a short pointed base, the middle section being the widest and capitate paraphyses. *Orbilia renispora* differs from *O. baisensis* by the smaller (3.0–3.6 × 1.5–1.8) and lower curl ascospores. *O. leucostigma* and *O. xanthostigma* differ from *O. baisensis* by the equant end of ascospores. In addition, molecular analyses reveal that *O. baisensis* shares less than 91.20% similarity with *O. renispora* in ITS sequence, and 89.91% similarity with *O. xanthostigma* and 89.53% similarity with *O. leucostigma*, respectively. Both the morphological and the molecular evidence support their separation at the species level.

**Figure 2:**
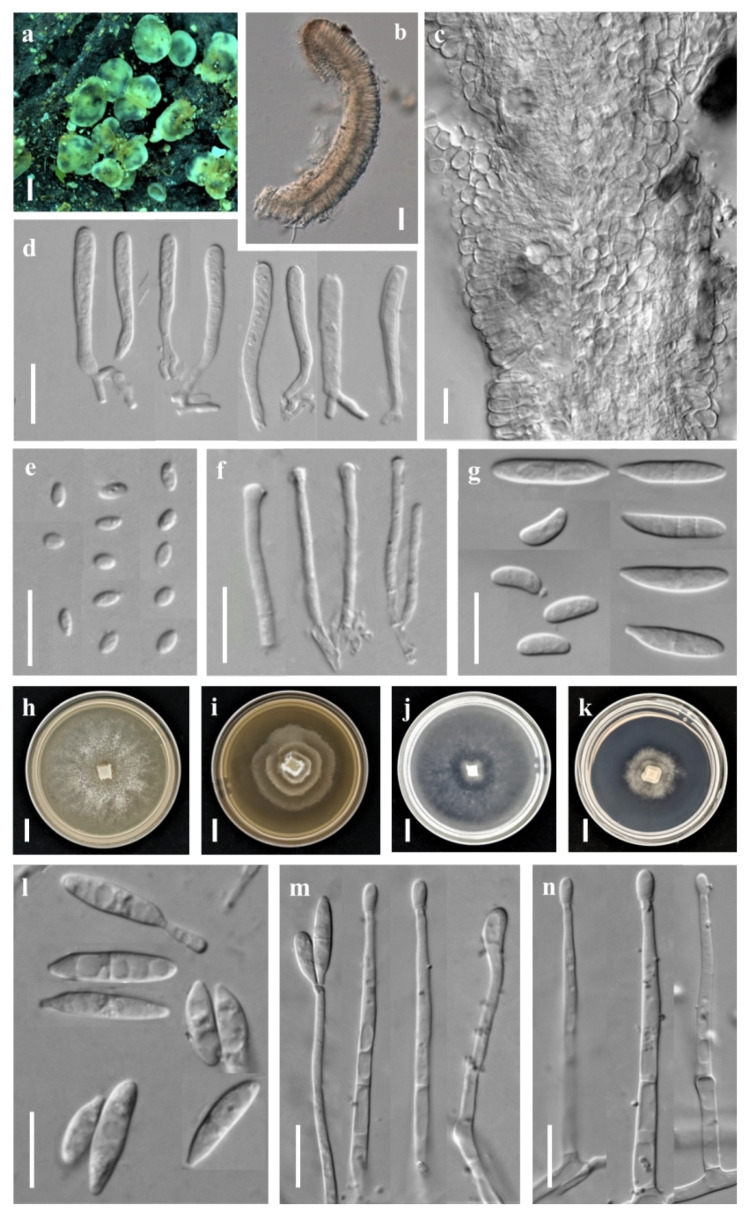
*Orbilia hanzhongensis* and *dactylella*-like asexual morph (strain BY35 isolated from GXU2365). (**a**) apothecia; (**b**) vertical section of apothecium; (**c**) basal excipular cells; (**d**) ascus; (**e**) ascospores; (**f**) paraphyses; (**h**–**k**) colony after 10 d at 25 °C, (**h**) on PDA, (**i**) on MEA, (**j**) on CMA, (**k**) on LY; (**g**,**l**) conidia; (**m**–**n**) conidiophores with conidia. Scale bars: (**a**) = 0.5 mm; (**b**) = 20 μm; (**c**–**g**,**l**–**n**) = 10 μm; (**h**–**k**) = 10 mm.

***Orbilia hanzhongensis*** X.Y. Ou & Bin Liu, sp. nov. (Figure 2)

MycoBank: MB 846094.

Etymology: according to the geographical origin, Hanzhong (Shaanxi).

Holotype: CHINA, Shaanxi province, Hanzhong city, Foping County, Wangjiawan, from fallen branch, 23 July 2017, X.Y. Ou, holotype GXU2365. Strain BY35 was isolated from GXU2365.

Sexual state: Apothecia rehydrated 0.1–1.5 mm diam., superficial on the deadwood, gregarious, waxy, medium translucent, smooth, disc round and slight to strongly concave, sometimes flat, margin not protruding, broadly sessile, pale to light yellow when fresh or rehydrated, yellow when dry. Asci †21.7–39.7 × 2.3–3.6 μm, cylindric-clavate, 8-spored, spores uniseriate, pars sporifera †20.5–30.7 μm long, truncate to hemispherical at the apex, the base with short to medium long, flexuous stalk, forked to L-, T-, H- or Y-shaped. Ascospores †2.5–4.4 × 1.7–2.2 μm, hyaline, non-septate, smooth, fusoid to ellipsoid, to ovoid, to lemon-shaped, one end subacute to acute, other end round or often acute, straight; SBs †0.2–0.4 μm diam., globose, at the end close to the wall in alive mature ascospores. Paraphyses straight to slightly capitate at the apex, †18.8–40.3 × 1.5–1.9 μm, the base branched and expanded to 2.0–3.0 µm in diameter at the apex. Hymenium 48.2–73.8 μm thick; medullary excipulum 16.6–22.2 μm thick, composed of medium dense textura intricata with inflated cells, sharply delimited. Ectal excipulum composed of textura globulosa-angularis from the base to the flanks, thin-walled, slightly gelatinized, 30.2–51.4 μm thick, cells †3.8–10.8 × 3.2–9.9 μm diam., ovate to spherical.

Asexual state: *Dactylella*-like.

Colonies beige-white on PDA, 50.5 mm diam. at 25 °C after 10 d, aerial hyphae dense; beige-white on MEA, 31.7 mm diam., aerial hyphae rarely sparse; white on CMA, 60 mm diam., aerial hyphae absent; white on LY, 22.7 mm diam., aerial mycelium absent. Hyphae hyaline, septate, branched, smooth, *1.0–3.5 μm wide. Conidiophores hyaline, erected, septate, unbranched, *10.6–72.8 μm long, *2.0–4.2 μm wide at the base and gradually tapering to *0.9–1.5 μm wide at the tip where bearing 1–3 apical spore. Conidia thallic, hyaline, unbranched, cylindric-ellipsoid (-clavate), obtuse at one end, truncate at the other end, straight when mature, slightly inflect when the immature, *10.1–20.3 × 2.6–4.2 μm, 1 septate.

Additional specimen examined: China, Shaanxi province, Hanzhong city, Foping County, Wangjiawan, from rotten branches, 23 July 2017, X.Y. Ou, GXU2379.

Notes: *Orbilia hanzhongensis* is characterized by ellipsoid to ovoid ascospores having one end subacute to acute, and mostly acute on the other end, globose SBs, capitate paraphyses. It is related to *O. rectispora* (Boud.) Baral and *O. xinjiangensis* (J. Chen, L.L. Xu, B. Liu and Xing Z. Liu) E. Weber, Baral and Helleman, but *O. rectispora* differs in having narrowly cylindrical to fusoid-clavate and larger ascospores (†5–9 × 0.9–1.2 µm), and *O. xinjiangensis* differs in distinctly larger spores (†7–9 × 1.8–2 µm) and larger conidia (*45–54 × 8–11 µm) with more septa [3]. Moreover, there is 51 bp (8.46%) divergence in the ITS region between *O. hanzhongensis* and *O. xinjiangensis*, and 90 bp (17.82%) divergence in the ITS region of *O. rectispora*. Both the morphology and DNA sequence data distinguish them as different species.

**Figure 3:**
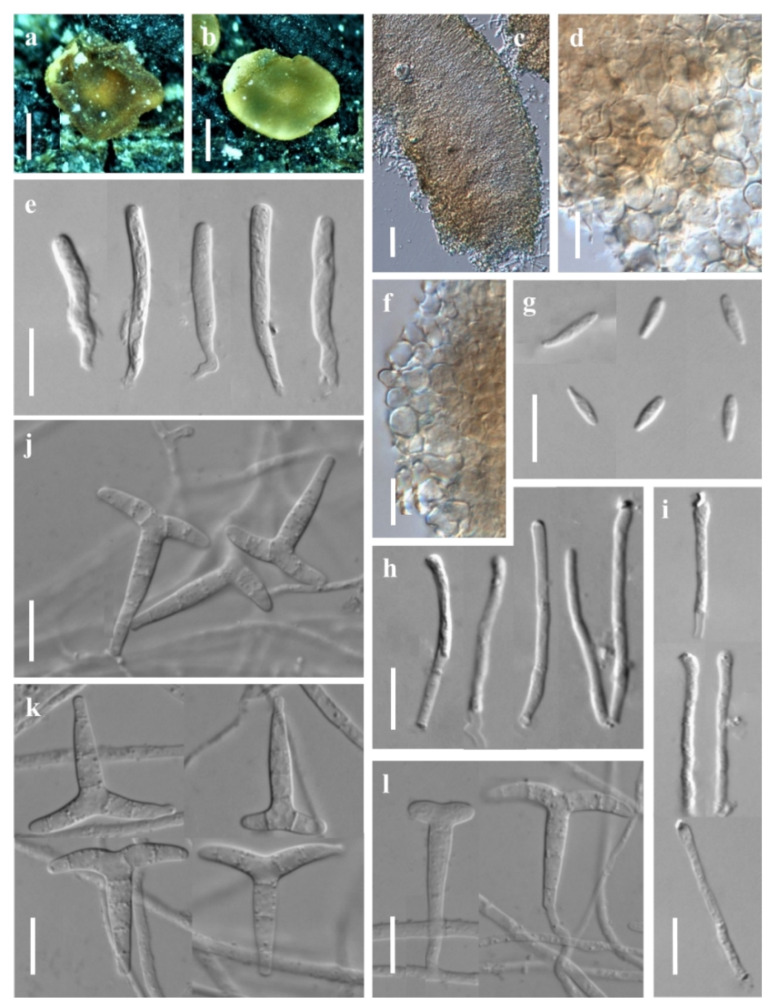
*Orbilia nanningensis* and *trinacrium*-like asexual morph (strain NN01 was isolated from GXU2466). (**a**,**b**) apothecia; (**c**) vertical section of apothecium; (**d**,**f**) basal excipular cells; (**e**) ascus; (**g**) ascospores; (**h**,**i**) paraphyses; (**j**,**k**) conidia; (**l**) conidiophores with conidia. Scale bars: (**a**,**b**) = 0.2 mm; (**c**) = 20 μm; (**d**–**l**) = 10 μm.

***Orbilia nanningensis*** X.Y. Ou & Bin Liu, sp. nov. (Figure 3).

MycoBank: MB 846095.

Etymology: name after the geographical origin, Nanning (Guangxi).

Holotype: CHINA, Guangxi province, Nanning city, Xixiangtang District, Shibu Town, from deadwood on the ground, 1 January 2020, X.Y. Ou, holotype GXU2466. Strain NN01 was isolated from GXU2466.

Sexual state: Apothecia rehydrated 0.2–0.5 mm diam., scattered, round, light yellow-orange, translucent, sessile, superficial on dead branches on the ground, orange when fresh, dry deep yellow to orange, disc strongly concave, wet light yellow, disc flat, smooth, margin thin or thick. Asci 20.9–55.7 × 2.4–4.6 μm, pars sporifera 14.0–37.2 μm, cylindric to clavate, 8-spored, the apex truncate to hemispherical, thin-walled, gradually narrowing towards the base, flexuous stalk, unforked. Ascospores †6.2–7.5 × 1.6–2.1 μm, clavate to fusoid with a distinct short tapered, rarely ellipsoid, one end obtuse, tapered 1.0–2.5 μm long, straight or slightly curved. Paraphyses straight to slightly capitate at the apex, †17.8–37.5 × 1.2–2.0 μm, basally unbranched and expanding to 1.9–3.2 µm in diameter, exudate 0.8–1.3 μm thick, over paraphyses. Hymenium 101.8–151.2 μm thick; medullary excipulum 56.8–79.0 μm thick, always composed of dense textura intricata with many inflated cells, sharply delimited. Ectal excipulum 53.6–87.4 μm thick, of thin-walled, composed of oriented textura globulosa-angularis from the base to the flanks or margin, cells †5.7–12.1 × 3.7–8.3 μm diameter.

Asexual state: *Trinacrium*-like.

Colonies white on PDA. Mycelium *1.7–3.2 μm wide. Conidiophores unbranched, erected or slightly bent, septate, *7.1–15.9 μm long, the base *1.8–2.0 μm wide, the tip *1.0–1.4 μm wide where bearing 1 apical spore. Conidia thallic, T-shaped, consisting of one stipe and two arms, the two arms bent downwards, total size *20.7–32.7 × 14.9–30.8 μm, the stipe *16.9–28.7 × 2.8–4.1 μm, 3–5 septate, the arms *6.0–13.8 × 2.3–3.6 μm, 1–3 septate.

Additional specimens examined: China, Guangxi province, Nanning city, Lewan farm, from deadwood, 1 January 2020, X.Y. Ou, GXU2467.

Notes: *Orbilia nanningensis* is clustered with *O*. cf. *paracaudata* Baral and G. Marson, *O. farnesianae* Baral, *O. pilifera* Baral and R. Galán, *O*. aff. *farnesianae* and *O*. *amarilla* Quijada and Baral. Their ascospores were fusoid to clavate with a short tapered, and with similar T- shaped of conidia, but *O. nanningensis* differs from other related species by smooth margin of apothecia and straight to slightly capitate paraphyses at the apex. Among the known species of *Orbilia*, *O*. cf. *paracaudata* is the most closely related to *O. nanningensis* in the phylogenetic tree, there is only a distance of 3.80% in the ITS region between *O. nanningensis* and *O*. cf. *paracaudata*. However, *O. paracaudata* can be separated from *O. nanningensis* by its longer and narrower ascospores (5.8–8.5 × 1.6–1.8 µm) and a distinctly protruding apothecial margin. 

**Figure 4:**
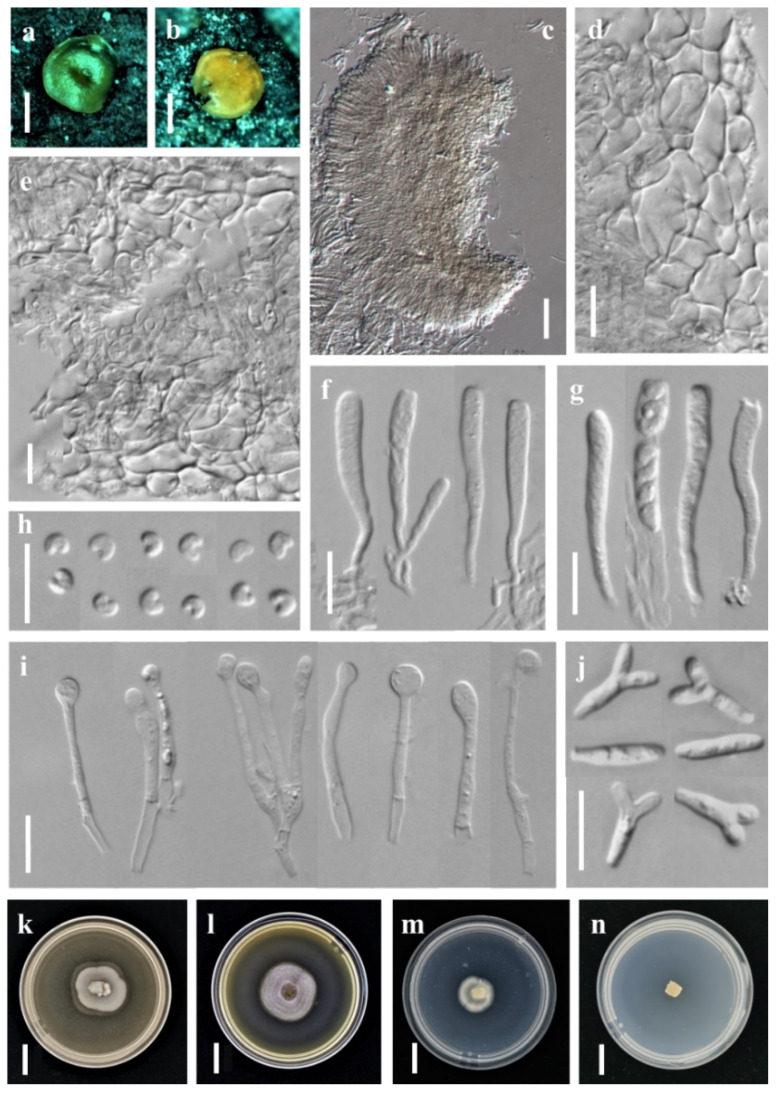
*Orbilia pinea* and *dicranidion*-like asexual morph (strain BY38 was isolated from GXU2368). (**a**,**b**) apothecia; (**c**) vertical section of apothecium; (**d**,**e**) basal excipular cells; (**f**,**g**) ascus; (**h**) ascospores; (**i**) paraphyses; (**j**) conidia; (**k**–**n**) colony at 25 °C, (**h**) on PDA after 10 d, (**i**) on MEA after 10 d, (**j**) on CMA after 30 d, (**k**) on LY after 30 d; Scale bars: (**a**,**b**) = 0.5 mm; (**c**–**j**) = 10 μm; (**k**–**n**) = 10 mm.

***Orbilia pinea*** X.Y. Ou & Bin Liu, sp. nov. (Figure 4).

MycoBank: MB 846096.

Etymology: named after the host from which it was collected, Pinus. 

Holotype: CHINA, Shaanxi province, Hanzhong city, Foping County, Wangjiawan, from deadwood of pinus on the ground, 23 July 2017, X.Y. Ou, holotype GXU2368. Strain BY38 was isolated from GXU2368.

Sexual state: Apothecia rehydrated 1.0 mm diam., yellowish to orange, translucent, round, superficial and scattered, waxy, smooth, disc flat, margin thin and not protruding, sessile, dry orange or honey-yellow when fresh. Asci †26.5–41.0 × 2.6–4.2 μm, cylindric-clavate, 8-spored, spores uniseriate, ~3-seriate, ~4 lower spores inverted (sometimes mixed), pars sporifera †16.9–26.8 μm long, the apex strongly truncate or round to hemispherical, the base gradually narrowing with short to medium long and flexuous stalk, forked to L-, H- or Y-shaped. Ascospores †2.5–3.3 × 1.5–2.2 μm, hyaline, non-septate, smooth, pronounced reniform, strongly curved, ~48–158°, end round, rarely obtuse, middle largest; SBs globose, †0.4–0.6 μm diameter, usually close to one end in alive mature ascospores. Paraphyses apically inflated to capitate at the apex, sometimes uninflated or slightly inflated to sublageniform, †17.3–45.3 × 1.3–2.8 μm, branched at the base and expanded to 2.3–5.4 µm in diameter at the apex. Hymenium 77.6–139.0 μm thick; medullary excipulum 46.6–65.0 μm thick, subhyaline, composed of dense loose textura intricata, sharply delimited. Ectal excipulum 53.2–92.4 μm thick, hyaline, composed of thin-walled, textura globulosa-angularis from the base to the margin, cells †6.2–22.2 × 5.0–13.7 μm diameter.

Asexual state: *Dicranidion*-like.

Colonies beige-white on PDA, 20.0 mm diam. at 25 °C after 10 d, aerial hyphae absent; beige-white on MEA, 23.7 mm diam., aerial hyphae sparse; grow very slowly on CMA, only 15 mm diam. at 25 °C after 30 d, and could not grow on LY. Hyphae hyaline, septate, branched, smooth. Conidiophores hyaline, erected or slightly bent, septate, unbranched at the base, the tip where bearing 1 apical spore. Conidia thallic, hyaline, Y-shaped, consisted of a stipe and two equal or unequal arms; the stipe *5.9–10.2 × 2.2–2.7 μm, 1 septate; the arms *2.1–4.5 × 1.6–2.4 μm, 1 septate; in addition, columnar conidia *13.4–13.7 × 2.5–2.7 μm, 1–3 septate.

Notes: *Orbilia pinea* is most similar to *O. fissilis* (K. Ando and Tubaki) E. Weber and Baral, the most remarkable feature of *O. pinea* is pronounced reniform and strongly curved ascospores, *O. fissilis* differs in broadly ellipsoid to subglobose ascospores and frequently 4-armed of the *Dicranidion*-like conidia. There is only a distance of 2.36% in the ITS region between *O. pinea* and the type strain of *O. fissilis*. Obviously, they are not conspecific.

#### 3.1.2. New Record Species

**Figure 5:**
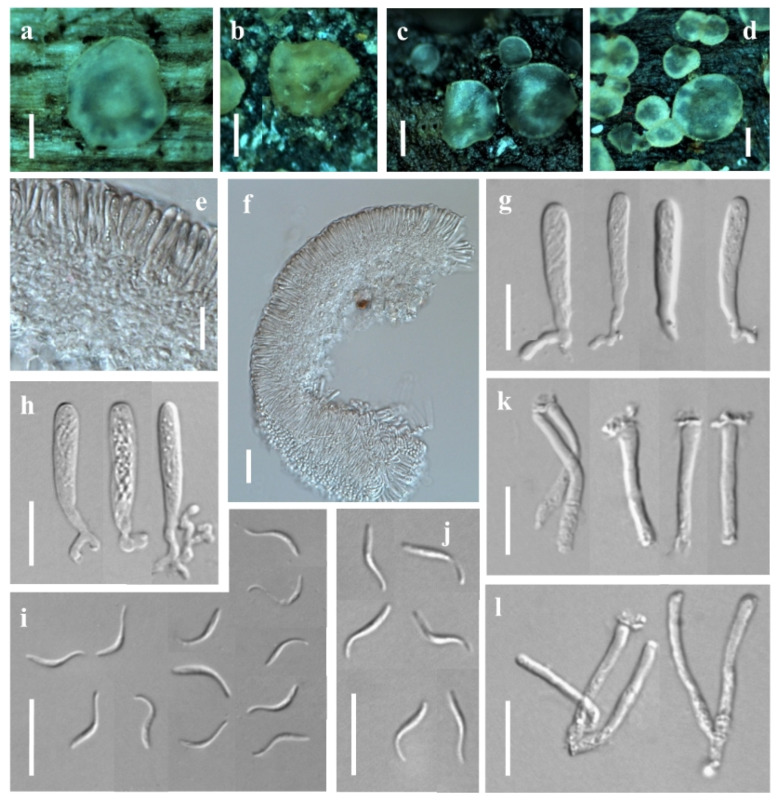
*Orbilia crenatomarginata* ((**a**,**e**,**f**,g,**j**,**k**–**l**) from GXU2342; (**b**,**c**) from GXU2343; (**d**,**h**,**i**) from GXU2383). (**a**–**d**) apothecia; (**e**,**f**) vertical section of apothecium; (**g**,**h**) ascus; (**i**,**j**) ascospores; (**k**,**l**) paraphyses. Scale bars: (**a**,**b**) = 0.2 mm; (**e**,**g**–**l**) = 10 μm; (**f**) = 20 μm.

***Orbilia crenatomarginata*** (Höhn.) Sacc. & Trotter, Syll. Fung. 22: 725 (1913) (Figure 5).

Sexual state: Apothecia 0.1–0.5 mm in diameter, scattered on the surface of rotten wood, superficial, flat or slightly convex, smooth, sessile, margin protruding (or denticulate) and filamentous, with small and distinct triangular teeth, dry pale or light yellow to cream-carneous, rehydrated and fresh greyish to white. Asci †20.8–37.5 × 2.6–3.9 μm, clavate, pars sporifera †11.4–19.7 μm, 8-spored, spores strongly spirally and closely twine within asci, truncate to hemispherical at the apex, base gradually narrowed, flexuous stalk, forked to L-, or Y-shaped. Ascospores †8.8–10.2 × 0.9–1.0 μm wide, hyaline, non-septate, helicoid or S-shaped, sickle-shaped or falculate from profile, cylindrical at the one end, tapered at the other end, the four lower spores inversely oriented; spore bodies tear-shaped. Paraphyses †15.1–37.7 × 1.5–2.1 μm, cylindrical to claviform or slightly capitate, unbranched or occasionally branched at the base, slightly enlarged at the apex, 1.7–3.1 µm, covered with waxy exudates, 0.5–1.3 μm thick. Hymenium 41.9–67.9 μm thick, ectal excipulum composed of textura globulosa-angularis.

Specimens examined: China, Shaanxi province, Baoji city, Meixian County, Taibai mountain forest park, from branch of deciduous tree lying on the ground, 21 July 2017, X.Y. Ou, GXU2342. China, Shaanxi province, Baoji city, Meixian County, Taibai mountain forest park, from branch of deciduous tree lying on the ground, 21 July 2017, X.Y. Ou, GXU2343. China, Shaanxi province, Ankang city, Ningshan County, Huoditang of Qinling, from branch of deciduous tree lying on the ground, 24 July 2017, X.Y. Ou, GXU2383.

Notes: *Orbilia crenatomarginata* features on strongly helicoid, worm or S-shaped ascospores, cylindrical but round gradually at the one end, strongly attenuated at the other end, cylindrical to claviform or slightly capitate paraphyses, apothecia margin denticulate with small and distinct triangular teeth. Our three specimens (GXU2342, GXU2343, GXU2383) corresponded to *O*. *crenatomarginata* H.B. 9452 and *O*. *crenatomarginata* H.B. 9265 (MLBP/BIPP = 100%/100%).

**Figure 6:**
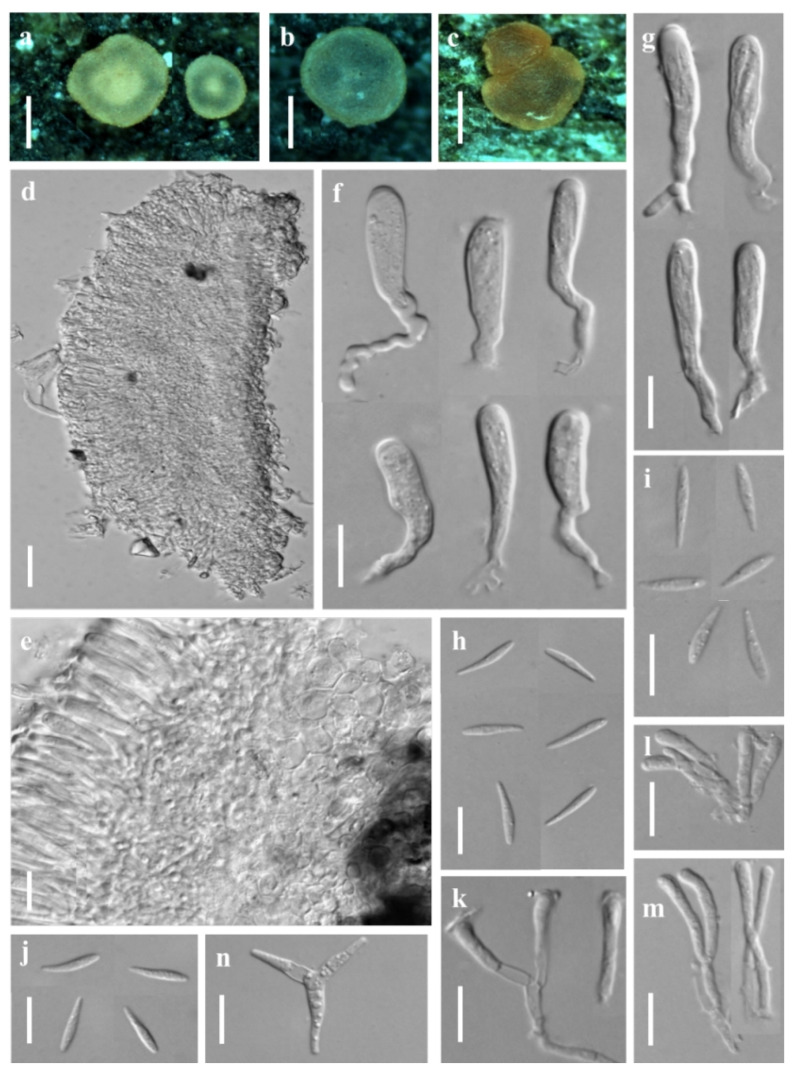
*Orbilia vinosa* ((**a**,**d**–**f**,**h**,**j**,**k**,**n**) from GXU2394; (**b**,**m**) from GXU2397; (**g**,**i**) from GXU2415; (**c**,**l**) from GXU2421). (**a**–**c**) apothecia; (**d**,**e**) vertical section of apothecium; (**f**,**g**) ascus; (**h**–**j**) ascospores; (**k**–**m**) paraphyses; (**n**) conidia (from apothecium). Scale bars: (**a**–**c**) = 0.2 mm; (**d**) = 20 μm; (**e**–**n**) = 10 μm.

***Orbilia vinosa*** (Alb. and Schwein.) P. Karst., Bidr. Känn. Finl. Nat. Folk 19: 101 (1871) (Figure 6).

Sexual state: Apothecia 0.1–0.4 mm in diam., scattered or gregarious on the surface of bark, disc flat to concave, sessile, waxy, translucent, round, fresh pale or light yellow to orange, sometimes cream-ochraceous or greyish, margin with crenulate, the back of disc with white glassy filament. Asci †16.2–53.4 × 5.5–6.0 μm, cylindric-clavate, pars sporifera †14.0–31.2 μm, 8-spores, spores seriate, lower spores inversely oriented, the apex hemispherical to truncate, the base gradually narrowing, flexuous stalk, forked to T-, L- or Y-shaped. Ascospores †7.3–14.1 × 1.1–2.3 μm, hyaline, non-septate, clavate, sometimes fusoid, one end obtuse or round, the other end slightly curved and smaller, strongly attenuated; spore bodies tear-shaped. Paraphyses †14.3–35.2 × 1.2–2.4 μm, cylindrical to slightly clavate-capitate, unbranched or occasionally branched at the base, slightly enlarged at the apex, terminal inflated, 1.9–3.3 μm in diameter. Hymenium 56.3–86.6 μm thick, ectal excipulum composed of textura globulosa-angularis, cell 3.5–10.2 × 2.4–8.0 μm and globose.

Specimens examined: China, Shaanxi province, Xi’an city, Cuihua Mountain, from rotten branch lying on the ground, 25 July 2017, X.Y. Ou, GXU2394. China, Shaanxi province, Xi’an city, Cuihua Mountain, from rotten wood lying on the ground, 25 July 2017, X.Y. Ou, GXU2397. China, Shaanxi province, Baoji city, Meixian County, Taibai mountain forest park, from branch of deciduous tree lying on the ground, 21 July 2017, X.Y. Ou and B. Liu, GXU2415. China, Shaanxi province, Hanzhong city, Foping County, Wangjiawan, from deadwood lying on the ground, 23 July 2017, X.Y. Ou and B. Liu, GXU2421.

Notes: *Orbilia vinosa* is characterized by clavate-fusoid ascospores, straight or slightly curved, one end obtuse, the other end strongly tapered. The gross morphology of our collections is similar to the original description, according to the detailed description and illustrations of the species provided by Baral et al. [3]. Sequence comparisons also revealed that the three specimens (GXU2394, GXU2397, GXU2421) corresponded to *O. vinosa* G.M. 2014-02-14 and *O. vinosa* CBS 116215 (MLBP/BIPP = 100%/100%).

**Figure 7:**
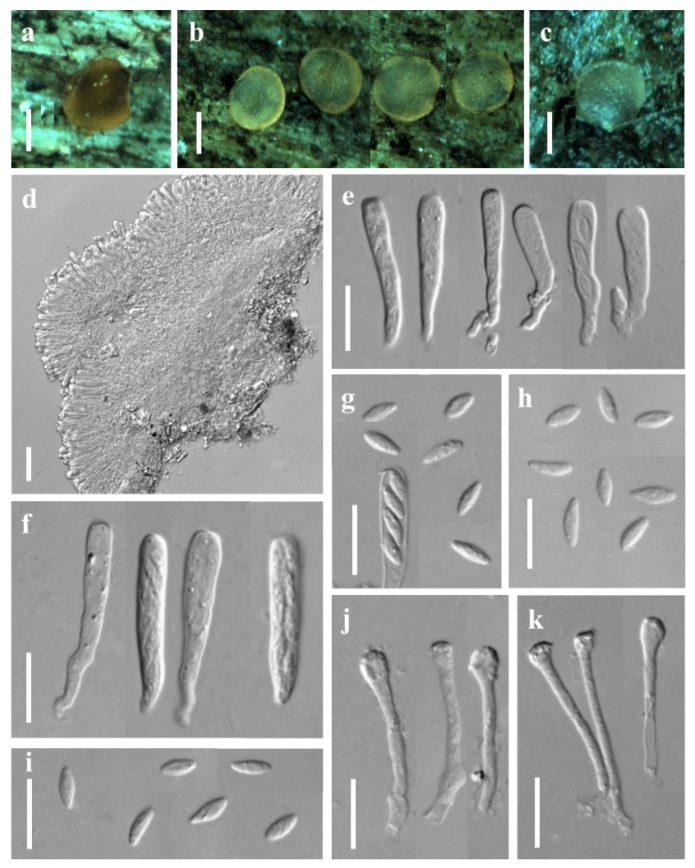
*Orbilia vitalbae* ((**a**,**b**,**d**,**e**,**h**,**j**) from GXU2438; (**c**,**f**,**g**,**i**,**k**) from GXU2442). (**a**–**c**) apothecia; (**d**) vertical section of apothecium; (**e**,**f**) ascus; (**g**–**i**) ascospores; (**j**,**k**) paraphyses. Scale bars: (**a**–**c**) = 0.2 mm; (**d**) = 20 μm; (**e**–**k**) = 10 μm.

***Orbilia vitalbae*** Rehm, in Ade, Hedwigia 64: 315 (1923) (Figure 7).

Sexual state: Apothecia 0.1–0.4 mm in diameter, superficial on the rotten branch, scattered or gregarious, disc flat or slightly convex, round, translucent, sessile, pale to yellowish when fresh or rehydrated, dry deep cream to orange-yellow, margin slightly crenulate. Asci †20.0–51.0 × 3.1–5.2 μm, pars sporifera †19.1–26.2 μm, cylindric-clavate, 8-spored, the apex obtuse or strongly truncate, the base gradually thin, flexuous stalk, the lower part bifurcate to L- or Y-shaped. Ascospores †5.1–7.7 × 1.8–2.5 μm, fusoid to clavate, one end round to obtuse or subacute, the other end gradually attenuated, solely fastigiate arrangement in the ascus; SBs tear-shaped, in the end of ascospores. Paraphyses †19.5–29.8 × 1.2–2.5 μm, capitate, unbranched, enlarged to globose at the apex, 2.4–4.5 μm, a waxy exudate over terminal cell of paraphyses. Hymenium 66.6–102.1 μm thick, ectal excipulum composed of textura globulosa-angularis.

Specimens examined: China, Shaanxi province, Xi’an city, Cuihua Mountain, from rotten branch lying on the ground, 25 July 2017, X.Y. Ou and B. Liu, GXU2438. China, Shaanxi province, Xi’an city, Cuihua Mountain, from deadwood lying on the ground, 25 July 2017, X.Y. Ou and B. Liu, GXU2442.

Notes: *Orbilia vitalbae* featured on unipolar and straight, fusoid to clavate ascospores, round to obtuse at the one end and attenuated at the other end. In this study, our two specimens (GXU2438, GXU2442) corresponded to *O*. *vitalbae* H.B. 9905a (MLBP/BIPP = 99%/100%).

### 3.2. Phylogenetic Analysis

**Figure 8:**
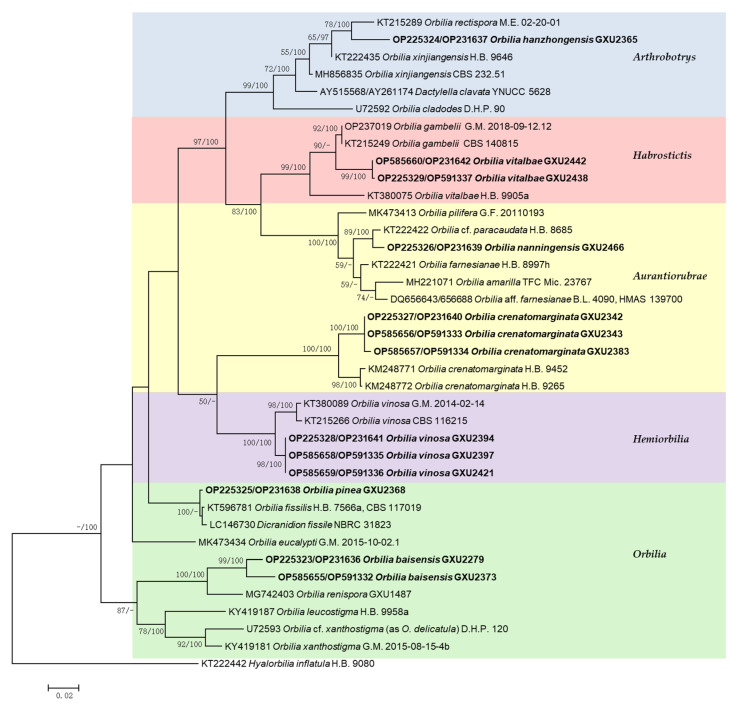
Phylogenetic tree generated from maximum likelihood analyses based on the combined ITS and LSU sequences expressing relationship of *Orbilia* species. Maximum likelihood bootstrap support ≥ 50% (left) and Bayesian posterior probability values ≥ 95% (right) are indicated at nodes (BIBP/MLBP). *Hyalorbilia inflatula* H.B. 9080 was used as outgroup. Bold names represent new species and new Chinese record.

The phylogenetic tree (Figure 8) was inferred from maximum likelihood analyses and Bayesian inference analyses with the combined ITS and LSU (528 bp from ITS and 561 bp from LSU) sequences. The analysis involved 38 nucleotide sequences that belonged to 25 species, 13 sequences were recognized in this study. The tree was composed of 37 strains as ingroup. *Hyalorbilia inflatula* (P. Karst.) Baral and G. Marson was used as the outgroup taxon. Maximum likelihood and Bayesian inference analyses generated semblable tree topologies. In the phylogenetic tree, five clades corresponding to sections of *Orbilia*, including *Arthrobotrys*, *Aurantiorubrae*, *Habrostictis*, *Hemiorbilia* and *Orbilia*, were revealed (Figure 8).

In the phylogenetic tree inferred from combined sequences, our 13 samples were considered as four new species and three new record species in *Orbilia*. The new species *Orbilia baisensis* were located in a clade with high statistical support (MLBP/BIPP = 100%/100%) with *O. renispora*. The two specimens (GXU2279, GXU2373) formed a subclade and designated as *O. baisensis* (MLBP/BIPP = 99%/100%). *Orbilia hanzhongensis* and *O. rectispora* received medium statistical support (MLBP/BIPP = 78%/100%). *Orbilia nanningensis* was related to *O*. cf. *paracaudata*, *O*. *nanningensis* and *O*. cf. *paracaudata* clustered together in a high supported subclade (MLBP/BIPP = 89%/100%). *Orbilia pinea* and *O. fissilis*, *Dicranidion fissile* were located in a clade with high support (MLBP = 100%, BIPP < 95%). *O. pinea* clustered with *O. fissilis* in a high support (MLBP = 100%, BIPP < 95%). 

New records species *Orbilia crenatomarginata* and *O. vinosa* clustered in a clade, which was divided into two strong supported monophyletic subclades. Our three specimens (GXU2342, GXU2343, GXU2383) formed a subclade corresponded to *O*. *crenatomarginata* (MLBP/BIPP = 100%/100%) and the three specimens (GXU2394, GXU2397, GXU2421) formed a subclade corresponding to *O. vinosa* (MLBP/BIPP = 100%/100%). *Orbilia vitalbae* and *O. gambelii* were located in a clade with high statistical support (MLBP/BIPP = 99%/100%). Our two specimens (GXU2438, GXU2442) formed a subclade corresponding to *O*. *vitalbae* (MLBP/BIPP = 99%/100%).

## 4. Discussion

The genus *Orbilia* is diversely and widely distributed in China. The morphological characteristic of the specific ascus and the polymorphic ascospores, especially the strongly refractive spore body, makes *Orbilia* distinctly unique to the other discomycetes. So far, only several species of *Orbilia* have been reported from Guangxi and Shaanxi province, China. In this study, sixteen specimens were collected from Guangxi and Shaanxi province, China. Seven species of *Orbilia* were identified based on morphological characters and phylogenetic analyses, containing four new species, viz. *Orbilia baisensis*, *O. hanzhongensis*, *O. nanningensis*, *O. pinea*, and three newly recorded species to China, viz. *O. crenatomarginata*, *O. vinosa* and *O. vitalbae*. Asexual states of the four new species are confirmed by obtaining pure cultures from the fresh apothecium, which connected to the anamorphic genus of *Dicranidion*, *Dactylella* and *Trinacrium*.

*Orbilia baisensis* is clustered with *O. renispora*, *O. leucostigma* and *O. xanthostigma* by having reniform to nephroid or C-shaped ascospores. *O. baisensis* features on 8-spored asci, flexuous base and forked to L-, T- or Y-shaped at the base, pronounced reniform ascospores, round and larger at the one end, small pointed base at the other end, the middle section being the widest and capitate paraphyses. *Orbilia renispora* differs from *O. baisensis* by the smaller and lower curl ascospores [18]. It can be confused with species of *O. xanthostigma***-***leucostigma* complex. However, *O. leucostigma* and *O. xanthostigma* differ from *O. baisensis* by the equant end of ascospores. Ascospores of *xanthostigma***-***leucostigma* complex are smaller, and with verrucose granule on the dorsal side [11,16]. The distinct warts on the dorsal side of ascospores were reported for the first time by Spooner [7], whereas Baral treated *O. delicatula* as the synonymy of *O.* cf. *xanthostigma* [3]. It was problematic to identify as *O. xanthostigma* and *O. leucostigma* only drawing on different color of apothecia by previous research, actually they contained different species, so they were arranged into *xanthostigma***-***leucostigma* complex. Baral revealed the high genovariation and represented multiple invariable genotypes in *Orbilia xanthostigma* and *Orbilia leucostigma* [23].

*Orbilia hanzhongensis* is characterized by fusoid to ellipsoid, to ovoid, to lemon-shaped ascospores with subacute to acute at the one end, round or often acute at the other end, globose SBs. It was related to *O. rectispora*, but differed in having ovoid-fusoid and smaller ascospores. Meanwhile, *O. hanzhongensis* deviated from *O. rectispora* [46] by a 9.47% distance in the ITS region. The sequences taken from the pure culture of *Orbilia nanningensis* comprised ITS and LSU regions, it was closed to *O.* cf. *paracaudata*, whereas *O. nanningensis* was deviated from *O*. cf. *paracaudata* [3] by a 5.8% distance in the ITS region and it had smooth margin of apothecia and capitate paraphyses. *Orbilia pinea* was related to *O. fissilis* and *D. fissile*, it differed from *O. fissilis* by smooth and pronounced reniform ascospores. *Orbilia crenatomarginata* was described and illustrated in detailed under the name of *Orbilia crystallina* [47]. The species is distinguished by white to cream apothecia and margin with the crystalline tooth, flexuous and forked to L-, or Y-shaped asci, helicoid or S-shaped to sickle-shaped ascospores.

*Orbilia vinosa* has been reported in Africa, America, Asia and Europe, growing on gymnosperms and angiosperms [16], but was first reported in China. *Peziza vinosa* is the primitive name of *O. vinosa* and described poorly [48], Spooner supplemented descriptions in detail and solved some problems with the type of *Peziza vinosa* [7]. *O. vinosa* clustered in a clade with *O. crenatomarginata*, but the former one differs by clavate ascospores. 

*Orbilia vitalbae* can grow on rotten branches of various trees (Clematis et al.), decayed wood or herbaceous plants (Sideritis et al.) [49], and it is illustrated by asci †(27–)30–50(–54) × (3.5–)4–5.3(–5.5) μm and ascospores †5–8(–10) × 1.4–1.6 or 1.8–2.5(–2.7) μm [3]. In this study, the sizes of asci (†20.0–51.0 × 3.1–5.2 μm) and ascospores (†5.1–7.7 × 1.8–2.5 μm), the shape of ascospores, are well in agreement with the previous findings of Rehm.

Members of *Orbilia* are often found on dead twigs and branches hanging on trees, distributed in tropical, subtropical and temperate regions. There are 470 species currently known in the family *Orbiliaceae* [3], of which more than 100 species have been reported in China. Surveys of fungal resources in various regions with different climates and geographic structures will improve our understanding of the species diversity of orbiliaceous fungi in the country. It is necessary to investigate fungal resources in various regions in the future.

## Figures and Tables

**Table 1:** GenBank accession numbers of taxa used in phylogenetic analyses.

Species	Strain Number	GenBank Accession Number	
		ITS	LSU
*Dactylella clavata*	YNUCC 5628	AY515568	AY261174
*Dicranidion fissile*	NBRC 31823	LC146730	LC146730
*Hyalorbilia inflatula*	H.B. 9080	KT222442	KT222442
*Orbilia amarilla*	TFC Mic. 23767	MH221071	MH221071
Orbilia baisensis *	**DL17 (GXU2279)**	**OP225323**	**OP231636**
Orbilia baisensis *	**BY44 (GXU2373)**	**OP585655**	**OP591332**
*Orbilia cladodes*	D.H.P. 90	U72592	U72592
Orbilia crenatomarginata **	**(GXU2342)**	**OP225327**	**OP231640**
Orbilia crenatomarginata **	**(GXU2343)**	**OP585656**	**OP591333**
Orbilia crenatomarginata **	**(GXU2383)**	**OP585657**	**OP591334**
*Orbilia crenatomarginata*	H.B. 9452	KM248771	KM248771
*Orbilia crenatomarginata*	H.B. 9265	KM248772	KM248772
*Orbilia eucalypti*	G.M. 2015-10-02.1	MK473434	MK473434
*Orbilia farnesianae*	H.B. 8997h	KT222421	KT222421
*Orbilia* aff. *farnesianae*	B.L. 4090 (HMAS 139700)	DQ656643	DQ656688
*Orbilia fissilis*	CBS 117019	KT596781	KT596781
*Orbilia gambelii*	CBS 140815	KT215249	KT215249
*Orbilia gambelii*	G.M. 2018-09-12.12	OP237019	OP237019
Orbilia hanzhongensis *	**BY35 (GXU2365)**	**OP225324**	**OP231637**
*Orbilia leucostigma*	H.B. 9958a	KY419187	KY419187
Orbilia nanningensis *	**NN01 (GXU2466)**	**OP225326**	**OP231639**
*Orbilia* cf. *paracaudata*	H.B. 8685	KT222422	KT222422
*Orbilia pilifera*	G.F. 20110193	MK473413	MK473413
Orbilia pinea *	**BY38 (GXU2368)**	**OP225325**	**OP231638**
*Orbilia rectispora*	M.E. 02-20-01	KT215289	KT215289
*Orbilia renispora*	GXU1487	MG742403	MG742404
Orbilia vinosa **	**(GXU2394)**	**OP225328**	**OP231641**
Orbilia vinosa **	**(GXU2397)**	**OP585658**	**OP591335**
Orbilia vinosa **	**(GXU2421)**	**OP585659**	**OP591336**
*Orbilia vinosa*	G.M. 2014-02-14	KT380089	KT380089
*Orbilia vinosa*	CBS 116215	KT215266	KT215266
Orbilia vitalbae **	**(GXU2438)**	**OP225329**	**OP231642**
Orbilia vitalbae **	**(GXU2442)**	**OP585660**	**OP591337**
*Orbilia vitalbae*	H.B. 9905a	KT380075	KT380075
*Orbilia xanthostigma*	G.M. 2015-08-15-4b	KY419181	KY419181
*Orbilia* cf. *xanthostigma*	D.H.P. 120	U72593	U72593
*Orbilia xinjiangensis*	CBS 232.51	MH856835	MH856835
*Orbilia xinjiangensis*	H.B. 9646	KT222435	KT222435

Note: * new species, ** new Chinese record, specimen numbers are shown in parentheses, sequences newly generated in this study are in bold.

## Data Availability

The sequencing data were submitted to GenBank.
